# P-1260. Beyond Plasma: Defining the Translation Value of Epithelial Lining Fluid Exposure Profiles for the Treatment of Pneumonia

**DOI:** 10.1093/ofid/ofaf695.1451

**Published:** 2026-01-11

**Authors:** Yakun Fu, Andrew J Fratoni, David P Nicolau

**Affiliations:** Hartford hospital, Hartford, CT; Hartford Hospital, Hartford, CT; Hartford Hospital, Hartford, CT

## Abstract

**Background:**

Pharmacodynamic (PD) targets based on plasma exposure are commonly used for pneumonia (PNA). However, the exposure required in plasma may differ at the target site of infection when considering drug penetration. This study defined plasma and pulmonary epithelial lining fluid (ELF) PD targets for meropenem (MEM), cefiderocol (FDC), levofloxacin (LVX), and tobramycin (TOB) against a challenge set of *Klebsiella pneumoniae* (KP) in a preclinical PNA model, and assessed the predicted CFU/lung response of average human exposures relative to susceptible, intermediate, and resistant (SIR) MICs.

**Table 1.**
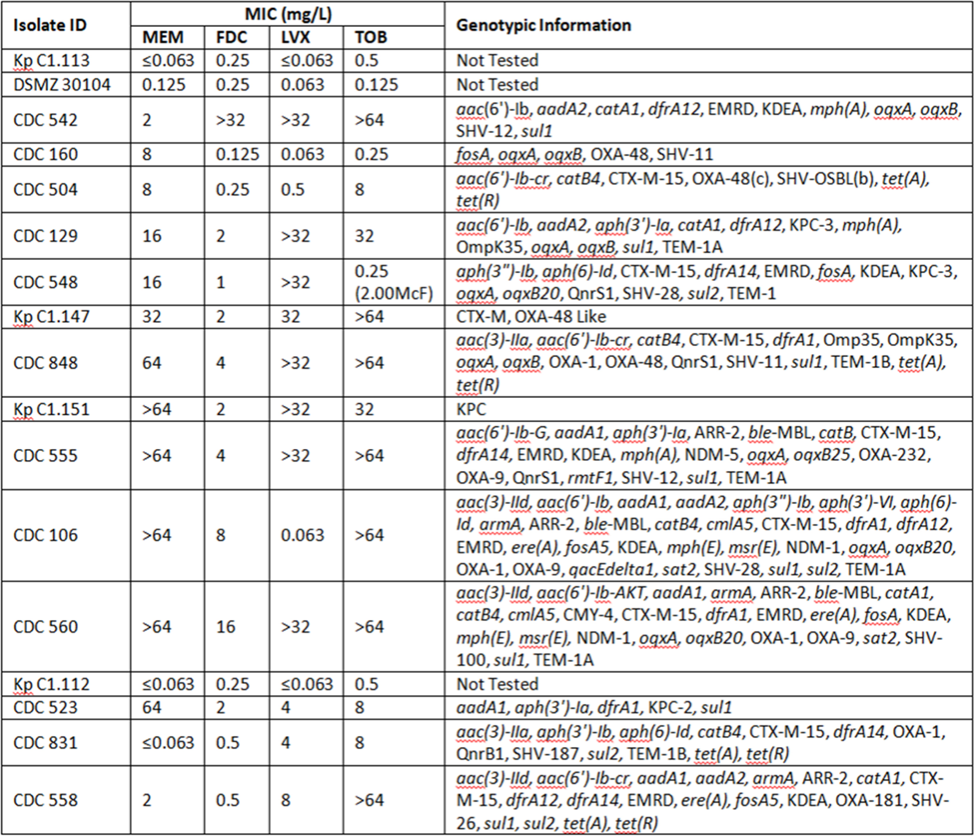
Phenotypic and genotypic information of Klebsiella pneumoniae isolates FDC, cefiderocol; LVX, levofloxacin; MEM, meropenem; MIC, minimum inhibitory concentration; TOB, tobramycin

**Table 2.**
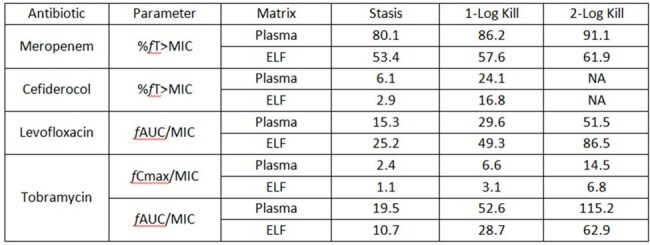
Plasma and ELF exposure targets in Klebsiella pneumoniae

**Methods:**

To provide a robust PD profile in both plasma and ELF, two dosing regimens per antibiotic were administered to neutropenic mice infected with 17 KPs (Table 1) following the COMBINE murine PNA protocol. Efficacy was evaluated as change in log_10_ CFU/lung at 24 h. Emax models were fitted to composite CFU data for each antibiotic, and plasma and ELF exposures required (%*f*T >MIC, *f*AUC/MIC, *f*Cmax/MIC) for stasis, 1- and 2-log_10_ reduction were calculated using the Hill equation. The average human plasma and ELF exposures achieved with MEM 2 g q8h over 3 h infusion, FDC 2 g q8h over 3 h infusion, LVX 750 mg q24h, and TOB 7 mg/kg were assessed relative to SIR breakpoints and predicted CFU/lung efficacy.

**Figure 1.**
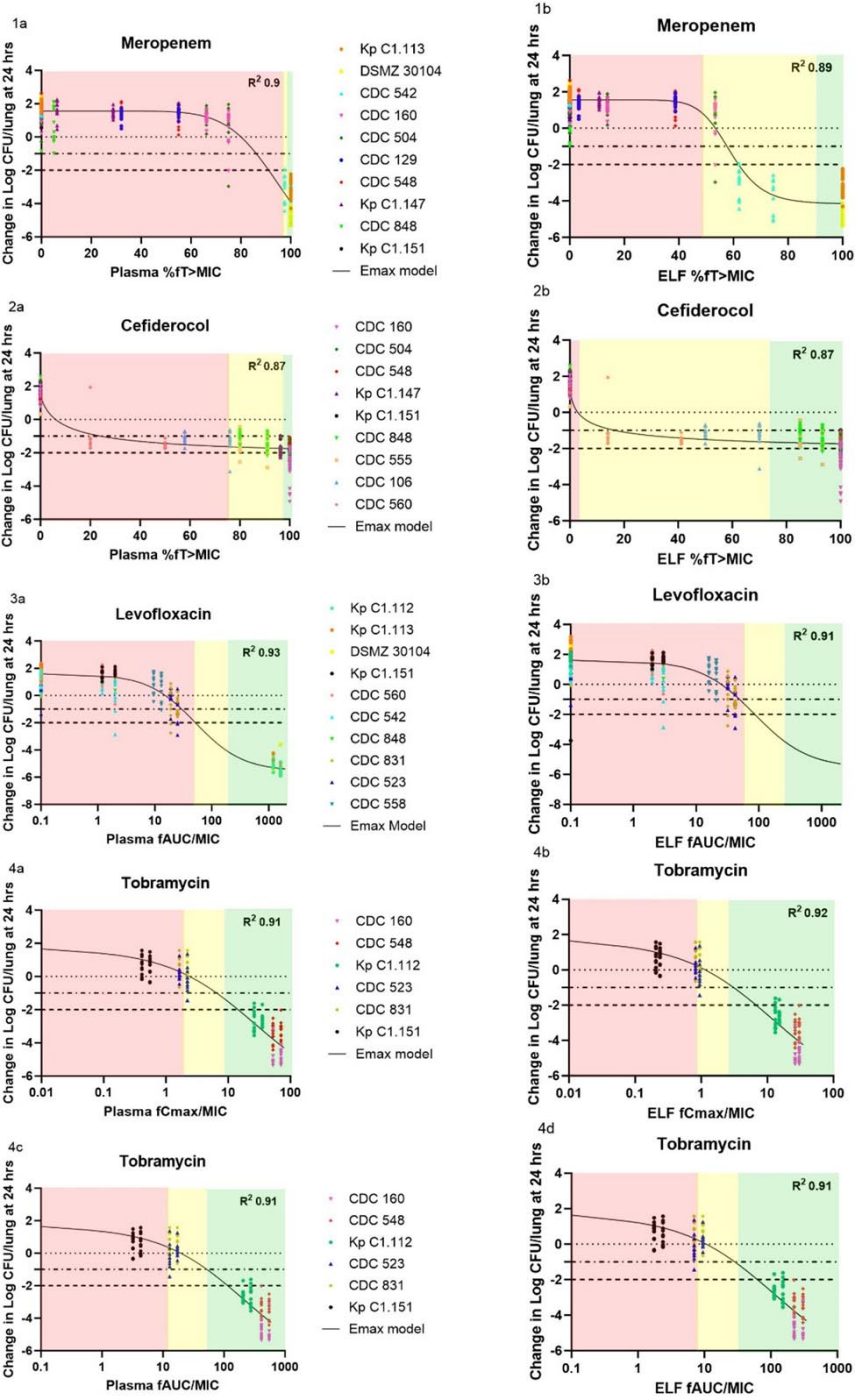
Plasma and ELF exposures of meropenem, cefiderocol, levofloxacin, and tobramycin versus CFU response against Klebsiella pneumoniae Green area: average free human exposures relative to susceptible MICs. Yellow area: average free human exposures relative to intermediate MICs. Red: average free human exposures relative to resistant MICs.

**Results:**

The ELF exposure required for stasis, 1-and 2-log_10_ CFU/lung reduction against KP were lower than in plasma for MEM, FDC, and TOB, but were higher than in plasma for LVX (Table 2/Figure 1). Notably, the plasma PD target for MEM was higher, and the magnitude of kill for FDC was lower relative to previously published data, as most KPs were carbapenemase producers. All Emax models had R^2^ > 0.85. At “S” MICs, the average human ELF exposure of all agents predicted a consistent >1 log_10_ CFU/lung reduction. The predicted CFU/lung responses at human ELF exposures were variable at “I” MICs, and limited at “R” MICs.

**Conclusion:**

The magnitude of free plasma and ELF exposures required to achieve PD targets were enumerated for four antibiotics of differing drug classes against KP. These data demonstrate the clinical translation of the model relative to established breakpoints and emphasize that exposures needed in plasma for efficacy may differ in magnitude in either direction from exposures needed at the target site of infection.

**Disclosures:**

Andrew J. Fratoni, PharmD, Qpex Biopharma, Inc.: Grant/Research Support David P. Nicolau, PharmD, CARB-X: Grant/Research Support|Innoviva: Advisor/Consultant|Innoviva: Grant/Research Support|Shionogi: Advisor/Consultant|Shionogi: Grant/Research Support

